# Development and validation of a new comorbidity index for patients with head and neck squamous cell carcinoma in Japan

**DOI:** 10.1038/s41598-017-07752-1

**Published:** 2017-08-04

**Authors:** Yukinori Takenaka, Norihiko Takemoto, Ryohei Oya, Naoki Ashida, Takahiro Kitamura, Kotaro Shimizu, Kazuya Takemura, Takahiro Michiba, Atsushi Hanamoto, Motoyuki Suzuki, Yoshifumi Yamamoto, Atsuhiko Uno, Hidenori Inohara

**Affiliations:** 1 Department of Otorhinolaryngology-Head and Neck Surgery, Osaka General Medical Center, Osaka, Japan; 2 Department of Otorhinolaryngology-Head and Neck Surgery, Osaka University Graduate School of Medicine, Osaka, Japan

## Abstract

Due to habitual drinking and smoking and advanced age at diagnosis, patients with head and neck squamous cell carcinoma (HNSCC) frequently present with comorbidities. Several comorbidity indices have been developed and validated for HNSCC. However, none have become the standard method. In this study, we developed a new comorbidity index for Japanese patients with HNSCC, which was validated against an independent data set. A Cox proportional hazards analysis of 698 patients identified dementia, connective tissue diseases, and second primary malignancies in the oesophagus, head and neck, lungs, and stomach as prognostic comorbidities for overall survival. The Osaka head and neck comorbidity index (OHNCI) was generated from the weighted points of these comorbidities. In the independent data set, the 5-year overall survival rates for the low, moderate, and high scoring OHNCI groups were 62.1%, 64.3%, and 37.7%, respectively. In the multivariate analysis, the high scoring OHNCI group was an independent prognostic factor for overall survival (hazard ratio: 1.81, 95% confidence interval: 1.05–3.13; P = 0.031). The model including the OHNCI exhibited a higher prognostic capability compared to those including other commonly used comorbidity indices. The OHNCI could become the primary choice for comorbidity assessment in patients with HNSCC in Japan.

## Introduction

Excessive smoking and drinking are the main aetiological causes of head and neck squamous cell carcinoma (HNSCC). Consequently, patients with HNSCC frequently present with comorbidities (e.g., cardiovascular disease, pulmonary disease, and diabetes)^1, 2^. Furthermore, as the entire aero-digestive tract may be exposed to carcinogens, these patients sometimes present with a prior history of cancer or concomitant second primary malignancies (SPMs)^3, 4^. The presence of concomitant SPMs is associated with an increased risk of cancer-related mortality owing to the difficulties in the simultaneous management of multiple cancers. Prior irradiation or surgery for metachronous HNSCC limits the treatment options for the current cancer. Non-cancer-related comorbidities cause non-cancer-related health events that result in non-cancer-related mortality^5^. In addition, a proportion of patients with severe comorbidities cannot tolerate an optimal cancer treatment, resulting in cancer-related mortality. Therefore, National Comprehensive Cancer Network guidelines recommend the documentation of comorbidities to facilitate optimal treatment selection^6^.

The established methods for evaluating comorbidities are required to incorporate comorbidity information into clinical decision-making. For such a purpose, several comorbidity indices have been developed to demonstrate comorbidity status. These comorbidity indices assign points to individual comorbid conditions, according to their severity or impact on mortality. Patients are then divided into groups with similar risk scores, according to the sum of their points. Among the indices, the Charlson comorbidity index (CCI), the Kaplan-Feinstein index (KFI), and their modifications have been widely used and validated in patients with HNSCC^2, 3, 7–11^. However, each comorbidity index is associated with its own advantages and disadvantages. Thus, none of these have become the standard method.

For use in a real clinical setting, a desirable comorbidity index for HNSCC should contain fewer items and be easily calculated. The index should be developed and validated specifically for patients with HNSCC because the frequency distribution and the impact of individual comorbid conditions on mortality may depend on the primary diseases. Furthermore, the incidence and distribution of comorbidities frequently observed in patients with HNSCC can vary quite considerably between different countries^1, 2, 7, 9, 12^ and their impact on prognosis may also differ. Therefore, a comorbidity index specifically developed for use in a particular region would be advantageous.

The aims of this study were to (1) determine the impact of individual comorbid conditions on the mortality of patients with HNSCC in Japan, (2) develop a new comorbidity index, (3) validate the prognostic capability of the index, and (4) compare its efficacy to that of other commonly used comorbidity indices.

## Results

### Characteristics of patients in the training set

The clinicopathological characteristics of the patients are summarised in Table 1. The male-to-female ratio was 5.1:1, with a median age of 66 (range, 21–92) years. The most common primary tumour site was the hypopharynx (n = 186; 26.6%), followed by the larynx (n = 161; 23.1%), oropharynx (n = 143; 20.5%), oral cavity (n = 119; 17.0%), and other (n = 89; 12.8%). Two hundred and forty-one patients (34.5%) had early-stage (Stage I–II) disease, while 437 patients (62.6%) had locoregionally advanced (Stage III–IVB) disease and 20 patients (2.9%) had metastatic (Stage IVC) disease. Curative treatment was administered to 628 patients (90.0%). Chemoradiotherapy and bioradiotherapy were the most preferred treatment options (n = 364; 52.2%), followed by surgery (n = 190; 27.2%). The performance status (PS) was good in 85% of the patients. The median follow-up duration of the surviving patients was 62.1 (range, 6.1–144.6) months.

**Table 1:** Characteristics of patients in the training (n = 698) and validation (n = 463) sets.

Characteristic	Training set (n = 698)	Validation set (n = 463)	P-value
Sex, n (%)			
M	584 (83.7)	337 (72.8)	
F	114 (16.3)	126 (27.2)	<0.001^*^
Age, years, median (range)	66 (21–92)	68 (27–92)	<0.001^*^
Primary tumour site, n (%)			
Hypopharynx	186 (26.6)	70 (15.1)	
Larynx	161 (23.1)	114 (24.6)	
Oropharynx	143 (20.5)	48 (10.4)	
Oral cavity	119 (17.0)	201 (43.4)	
Other	89 (12.8)	30 (6.5)	<0.001^*^
cStage, n (%)			
I	111 (15.9)	127 (27.4)	
II	130 (18.6)	73 (15.8)	
III	139 (19.9)	76 (16.4)	
IVA	252 (36.1)	162 (35.0)	
IVB	46 (6.6)	19 (4.1)	
IVC	20 (2.9)	6 (1.3)	<0.001^*^
Treatment intent, n (%)			
Curative	628 (90.0)	399 (86.2)	
Palliative	70 (10.0)	64 (13.8)	0.062
Treatment modality, n (%)			
Surgery	190 (27.2)	243 (52.5)	
RT	100 (14.3)	59 (12.7)	
CRT/BRT	364 (52.2)	113 (24.4)	
Chemotherapy	21 (3.0)	15 (3.3)	
Supportive care	23 (3.3)	33 (7.1)	<0.001^*^
Karnofsky performance status			
100–80	593 (85.0)	314 (67.8)	
70–50	98 (14.0)	127 (27.4)	
40–0	7 (1.0)	22 (4.8)	<0.001^*^

*P < 0.05.

BRT, bioradiotherapy; CRT, chemoradiotherapy; cStage, clinical stage; F, female; M, male; RT, radiotherapy.

### Prevalence of comorbidities

A summary of the prevalence of comorbidities in the training set is provided in Table 2. Synchronous and metachronous SPMs were common comorbidities, with overall prevalences of 13.2% and 15.8%, respectively. Among these patients, 11 patients had two synchronous SPMs, and 12 patients had two metachronous SPMs. Oesophageal cancer (OC) was the most prevalent synchronous SPM (n = 48 patients; 6.9%), followed by other (n = 21 patients; 3.0%) and gastric cancer (GC) (n = 19 patients; 2.7%). In contrast, head and neck cancer (HNC) (n = 33 patients; 4.7%) and OC (n = 32 patients; 4.6%) were the most prevalent metachronous SPMs. Several non-cancer-related comorbidities were also observed. Of these, diabetes (n = 98 patients; 14.0%), peptic ulcer disease (n = 44 patients; 6.3%), cerebrovascular disease (n = 41 patients; 5.9%), and chronic pulmonary disease (n = 40 patients; 5.7%) were the most frequently observed.

**Table 2:** Prevalence of comorbidities in patients with head and neck squamous cell carcinoma in the training set.

Comorbidity	Patients, n (%)
Synchronous SPM	92 (13.2)
Gastric cancer	19 (2.7)
Head and neck cancer	9 (1.3)
Lung cancer	6 (0.8)
Oesophageal cancer	48 (6.9)
Other	21 (3.0)
Metachronous SPM	110 (15.8)
Gastric cancer	19 (2.7)
Head and neck cancer	33 (4.7)
Lung cancer	8 (1.1)
Oesophageal cancer	32 (4.6)
Other	30 (4.3)
Diabetes	98 (14.0)
Peptic ulcer disease	44 (6.3)
Cerebrovascular disease	41 (5.9)
Chronic pulmonary disease	40 (5.7)
Peripheral vascular disease	20 (2.9)
Liver disease	33 (4.7)
Mild	21 (3.0)
Moderate/severe	12 (1.7)
Cardiac arrhythmia	18 (2.6)
Renal disease	16 (2.3)
Myocardial infarction	10 (1.4)
Connective tissue disease	8 (1.1)
Psychiatric disorders	7 (1.0)
Congestive heart failure	5 (0.7)
Dementia	4 (0.6)

SPM, secondary primary malignancy.

### Comorbidities and overall survival

Overall survival (OS) rates with or without individual comorbidities were estimated using Kaplan-Meier estimates, and evaluated with the Wilcoxon test (Table 3). Patients with synchronous lung cancer (LC), synchronous/metachronous OC, connective tissue disease, and dementia were associated with a significantly poorer OS compared to those without each of these diseases.

**Table 3:** Impact of comorbidities on 5-year overall survival (OS) in patients with head and neck squamous cell carcinoma in the training set.

Comorbidity		5-year OS rate (%) (95% CI)			P-value
Synchronous SPM	Y/N	42.5	/	62.8	<0.001^*^
		(31.5–53.1)		(58.6–66.7)	
Gastric cancer	Y/N	52.1	/	60.4	0.143
		(28.0–71.6)		(56.4–64.2)	
Head and neck cancer	Y/N	66.7	/	60.2	0.824
		(28.2–87.8)		(56.2–63.9)	
Lung cancer	Y/N	33.3	/	60.4	0.016^*^
		(4.6–67.6)		(56.4–64.1)	
Oesophageal cancer	Y/N	34.8	/	61.9	0.002^*^
		(20.4–49.6)		(57.9–65.7)	
Other	Y/N	50.6	/	60.6	0.084
		(27.1–70.0)		(56.5–64.2)	
Metachronous SPM	Y/N	50.6	/	61.9	0.015^*^
		(40.1–60.3)		(57.6–65.9)	
Gastric cancer	Y/N	50.3	/	60.4	0.152
		(26.2–70.3)		(56.4–64.2)	
Head and neck cancer	Y/N	37.5	/	61.0	0.108
		(16.1–59.0)		(57.0–64.7)	
Lung cancer	Y/N	71.4	/	60.0	0.495
		(25.8–92.0)		(56.1–63.8)	
Oesophageal cancer	Y/N	49.6	/	60.7	0.020^*^
		(31.4–65.4)		(56.7–64.5)	
Other	Y/N	62.7	/	60.1	0.844
		(42.8–77.4)		(56.0–63.9)	
Diabetes	Y/N	62.8	/	59.8	0.844
		(51.2–72.4)		(55.5–63.7)	
Peptic ulcer disease	Y/N	49.8	/	60.8	0.346
		(33.2–64.3)		(56.8–64.6)	
Cerebrovascular disease	Y/N	56.7	/	60.3	0.897
		(37.9–71.7)		(56.3–64.1)	
Chronic pulmonary disease	Y/N	57.2	/	60.3	0.074
		(39.8–71.2)		(56.3–64.1)	
Peripheral vascular disease	Y/N	53.0	/	60.4	0.904
		(27.0–73.5)		(56.4–64.1)	
Liver disease			/		
Mild	Y/N	66.7	/	59.9	0.960
		(42.5–82.5)		(55.9–63.7)	
Moderate/severe	Y/N	46.0	/	60.4	0.482
		(13.1–74.3)		(56.4–64.1)	
Cardiac arrhythmia	Y/N	73.2	/	59.9	0.225
		(43.0–89.1)		(55.9–63.6)	
Renal disease	Y/N	53.6	/	60.3	0.316
		(26.5–74.6)		(56.3–64.0)	
Myocardial infarction	Y/N	48.0	/	60.4	0.889
		(16.1–74.5)		(56.4–64.1)	
Connective tissue disease	Y/N	37.5	/	60.5	0.008^*^
		(8.7–67.4)		(56.5–64.2)	
Psychiatric disorders	Y/N	42.9	/	60.3	0.263
		(9.8–73.4)		(56.4–64.1)	
Congestive heart failure	Y/N	20.0	/	60.6	0.431
		(8.4–58.2)		(56.7–64.3)	
Dementia	Y/N	25.0	/	60.4	<0.001^*^
		(0.9–66.5)		(56.5–64.1)	

*P < 0.05.

CI, confidence interval; N, no; Y, yes.

### Development of the Osaka head and neck comorbidity index

To identify prognostic comorbidities, a Cox proportional hazard model was constructed using a stepwise method (Table 4). Covariates included in the initial model were all the comorbidities in Table 2, age, clinical stage, tumour site, sex and PS. The final model included age, clinical stage, tumour site, PS and the following comorbidities: synchronous LC (hazard ratio [HR]: 3.73, 95% confidence interval [CI]: 1.34–10.35; P = 0.012), synchronous OC (HR: 1.65, 95% CI: 1.08–2.51; P = 0.021), metachronous GC (HR: 1.73, 95% CI: 0.87–3.43), metachronous HNC (HR: 1.62, 95% CI: 0.95–2.76; P = 0.079), metachronous OC (HR: 1.58, 95% CI: 0.95–2.62; P = 0.078), connective tissue disease (3.89, 95% CI: 1.55–9.72; P = 0.004), and dementia (5.60, 95% CI: 1.44–21.69; P = 0.013). Comorbidities with a coefficient (log HR) of approximately 0.5 were each assigned 1 point. Then, synchronous LC, connective tissue disease, and dementia, with coefficients of approximately 1.5 for each, were each assigned 3 points. Thus, the sum of the points in each patient (range, 0–13) was determined as the Osaka head and neck comorbidity index (OHNCI) score.

**Table 4:** Cox proportional hazards analysis of comorbidities as prognostic factors for overall survival in patients with head and neck squamous cell carcinoma in the training set.

Comorbidity	HR (95% CI)^a^	P-value	Coefficient	Weighting
Synchronous SPM				
Lung cancer	3.73 (1.34–10.35)	0.012^*^	1.314	3
Oesophageal cancer	1.65 (1.08–2.51)	0.021^*^	0.498	1
Metachronous SPM				
Gastric cancer	1.73 (0.87–3.43)	0.116	0.548	1
Head and neck cancer	1.62 (0.95–2.76)	0.079	0.479	1
Oesophageal cancer	1.58 (0.95–2.62)	0.078	0.457	1
Connective tissue disease	3.89 (1.55–9.72)	0.004^*^	1.357	3
Dementia	5.60 (1.44–21.69)	0.013^*^	1.722	3

*P < 0.05.

^a^HRs were adjusted for age, primary tumour site, sex, Karnofsky performance status, and clinical stage.

CI, confidence interval; HR, hazard ratio; SPM, second primary malignancy.

### External validation of the Osaka head and neck comorbidity index and a comparison with other comorbidity indices

An independent data set was used to validate the prognostic significance of the OHNCI. The clinicopathological characteristics of the patients in the training and validation sets differed quite considerably (Table 1). In particular, the validation set included greater numbers of female patients, surgically treated patients, patients with poor PS, and patients with the oral cavity as the primary tumour site. In the validation set, the median follow-up duration of the surviving patients was 60.6 (range, 6.9–129.9) months. Patients were stratified into low, moderate, and high comorbidity scoring groups, according to each of the five different comorbidity indices (i.e., the CCI, the updated CCI, the head and neck-CCI [HN-CCI], the Washington University head and neck comorbidity index [WUHNCI], and the OHNCI) (Table 5).

**Table 5:** Relationship between different comorbidity indices and overall survival (OS) in the external validation set.

Comorbidity index	Patients n (%)	5-year OS rate (%)	95% CI	P-value	5-year cumulative incidence rate (%)					
					Index cancer-related mortality	95% CI	P-value	other cause mortality	95% CI	P-value
CCI score										
0	223 (48.2)	65.1	58.1–71.2		28.0	22.0–34.2		6.9	3.9–11.1	
1, 2	202 (43.6)	57.9	50.5–64.6		29.3	23.0–35.9		12.7	8.4–18.0	
≥3	38 (8.2)	49.7	30.9–65.9	0.101	31.3	16.2–47.6	0.693	19.1	7.2–35.1	0.007^*^
Updated CCI										
0	296 (63.9)	62.5	56.5–68.0		29.2	23.9–34.6		8.3	5.4–11.9	
1–2	133 (28.7)	59.6	50.2–67.7		27.8	20.3–35.7		12.7	7.3–19.5	
≥3	34 (7.3)	50.2	30.4–67.1	0.124	29.1	13.7–46.5	0.995	20.7	7.9–37.5	0.001^*^
HN-CCI score										
0	313 (67.6)	64.1	58.2–69.4		26.3	21.4–31.4		9.6	6.5–13.5	
1	133 (28.7)	54.7	45.5–63.0		32.7	24.6–41.0		12.6	7.5–19.1	
≥2	17 (3.7)	49.3	23.4–70.8	0.039^*^	43.7	18.5–66.6	0.125	7.0	0.3–28.8	0.237
WUHNCI score										
0	321 (69.3)	64.5	58.7–69.6		27.8	22.9–33.0		7.7	5.0–11.2	
1	85 (18.4)	61.3	49.1–70.4		27.7	18.2–37.9		11.1	5.0–19.9	
≥2	57 (12.3)	39.2	26.0–52.2	<0.001^*^	35.9	23.4–48.6	0.442	24.9	13.9–37.5	<0.001^*^
OHNCI score										
0	389 (84.0)	62.1	56.8–66.9		28.6	24.1–33.3		9.3	6.5–12.6	
1	47 (10.2)	64.3	48.4–76.5		26.8	14.7–40.4		8.9	2.8–19.5	
≥2	27 (5.8)	37.7	19.0–56.3	0.001^*^	33.3	16.4–51.3	0.719	29.0	12.0–48.4	<0.001^*^

*P < 0.05.

CCI, Charlson comorbidity index; HN, head and neck; OHNCI, Osaka head and neck comorbidity index; WUHNCI, Washington University head and neck comorbidity index.

**Figure 1:**
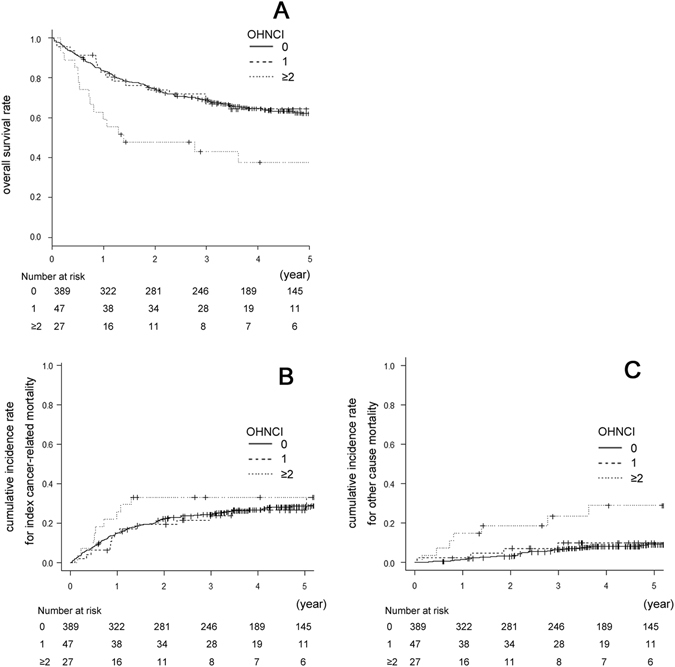
(**A**) Kaplan Meier estimates of overall survival according to OHNCI score. (**B**) Cumulative incidence rates for index cancer-related mortality according to OHNCI score. (**C**) Cumulative incidence rates for other cause mortality according to OHNCI score.

Figure 1 shows the overall survival rate, the cumulative incidence rate for index cancer-related mortality, and the cumulative incidence rate for other cause mortality according to OHNCI. The low, moderate, and high scoring OHNCI groups exhibited 5-year OS rates of 62.1%, 64.3%, and 37.7%, respectively (P = 0.001; Fig. 1A). Furthermore, the HN-CCI and the WUHNCI also exhibited significant associations with OS (P = 0.039 and P < 0.001, respectively; Table 5). There was an association between the occurrence of death and increased OHNCI, and WUHNCI scores (P = 0.029 and 0.029, respectively). However, those trends were not observed in the CCI, updated CCI, or HN-CCI (P = 0.660, 0.160 and 0.062, respectively). The 5-year cumulative incidence rates for index cancer-related mortality in the low, moderate, and high scoring OHNCI groups were 28.6%, 26.8%, and 33.3%, respectively (P = 0.719; Fig. 1B). Similarly, the other four comorbidity indices were not associated with index cancer-related mortality (the CCI [P = 0.693], the updated CCI [P = 0.995], the HN-CCI [P = 0.125], and the WUHNCI [P = 0.442]). The 5-year cumulative incidence rates for other cause mortality in the low, moderate, and high scoring OHNCI groups were 9.3%, 8.9%, and 29.0%, respectively (P < 0.001; Fig. 1C). Similarly, the CCI, the updated CCI and the WUHNCI were also significantly associated with other cause mortality (P = 0.007, 0.001 and P < 0.001, respectively). Collectively, the OHNCI was significantly associated with OS and other cause mortality, but was not associated with index cancer-related mortality. The impact of the OHNCI on mortality was considered to be comparable to that of the other comorbidity indices in the univariate analysis.

To further investigate the prognostic capability of these comorbidity indices, multivariate analysis was performed. Six Cox proportional hazards models were generated using the external validation data set: a baseline model with sex, age, primary tumour site, Karnofsky PS and clinical stage included as covariates and five models, which additionally included each of the five comorbidity indices (Table 6). The high scoring OHNCI group was an independent prognostic factor for OS (HR: 1.81, 95% CI: 1.05–3.13; P = 0.031). None of the other comorbidity indices were independent prognostic factors for OS. The Harrell’s concordance index (c-index) and Akaike information criterion (AIC) in the baseline and OHNCI models were 0.743 and 0.749, and 2110 and 2109, respectively. Notably, the OHNCI model exhibited the highest c-index and the lowest AIC across the models, indicating that the OHNCI was the most robust index among the five comorbidity indices examined.

**Table 6:** Cox proportional hazards model with each comorbidity index used as a covariate in the external validation set.

Comorbidity index	HR (95% CI)	P-value	c-index	AIC
Baseline model^a^	—	—	0.743	2110
CCI score				
0	Ref.	—	0.743	2112
1, 2	0.91 (0.66–1.26)	0.579	—	—
≥3	1.35 (0.79–2.23)	0.261	—	—
Updated CCI score				
0	Ref.	—	0.744	2112
1, 2	0.96 (0.69–1.33)	0.830	—	—
≥3	1.12 (0.63–1.87)	0.695	—	—
HN-CCI score				
0	Ref.	—	0.744	2114
1	1.03 (0.75–1.42)	0.842	—	—
≥2	1.29 (0.61–2.47)	0.487	—	—
WUHNCI score				
0	Ref.	—	0.745	2110
1	1.03 (0.69–1.49)	0.890	—	—
≥2	1.52 (1.00–2.25)	0.050	—	—
OHNCI score				
0	Ref.	—	0.749	2109
1	0.98 (0.60–1.59)	0.925	—	—
≥2	1.81 (1.05–3.13)	0.031^*^	—	—

*P < 0.05.

^a^Baseline model included age, sex, primary tumour site, and clinical stage and Karnofsky performance status.

AIC, Akaike information criterion; CCI, Charlson comorbidity index; CI, confidence interval; c-index, Harrell’s concordance index; HN, head and neck; HR, hazard ratio; OHNCI, Osaka head and neck comorbidity index; Ref., reference; WUHNCI, Washington University head and neck comorbidity index.

Finally, we conducted competing risk regression analyses for other cause related-mortality. After adjustment for age, sex and PS, the adjusted subdistribution HRs (sHRs) for other cause mortality were 0.96 (95% CI: 0.0.40–2.23: P = 0.920) for the moderate scoring OHNCI group and 2.88 (95% CI: 1.44–5.75: P = 0.003) for the high scoring OHNCI group.

## Discussion

Patients with HNSCC frequently present with comorbidities due to their elderly age and smoking and drinking habits. However, patients with severe comorbidities are not usually selected for clinical trials. Consequently, the outcomes of clinical trials cannot be directly applied to a community setting. To capitalise on the knowledge gained from clinical trials and to identify an optimal treatment approach for individual patients, it is essential to understand the impact of comorbidities on the treatment and prognosis of HNSCC. General comorbidity indices have been developed to evaluate comorbidities and stratify patients^7, 10^. Of these, the CCI, the KFI, and their modifications have been widely used for the risk stratification of patients with HNSCC^2, 3, 8, 9^. The CCI was generated from the data of patients admitted to the medical services of a teaching hospital^7^. The 19 comorbid conditions associated with 1-year mortality were assigned weighted scores of 1–6 points and the sum of the points was used as the comorbidity index. The KFI was established from data on the association between comorbidities in patients with diabetes and 5-year mortality^10^. These comorbidity indices are still used in their original form. However, modified versions have also been developed for specific diseases in order to more definitively discern the low- and high-risk groups. Through modification of the CCI, the HN-CCI was developed using Danish patients treated with radiotherapy^2^. In the HN-CCI, the 6 comorbid conditions that were prevalent in patients with HNSCC, and were associated with the prognosis of HNSCC, were identified and each were assigned 1 point. The HN-CCI was more efficient at delineating the high-risk group compared to the CCI. The WUHNCI was developed from the KFI^3^. The WUHNCI includes 7 items, with each item having a weighting of 1–4 points, according to its impact on 5-year survival. The adult comorbidity index-27 (ACE-27) is another comorbidity index for cancer patients based on the KFI, which has been validated for use in HNSCC^8^. The ACE-27 includes 27 items, with each item graded as mild, moderate, or severe. The ACE-27 was demonstrated to be more efficient than the WUHNCI for discerning the poor prognosis group in a cohort of elderly patients with HNSCC^13^. However, the ACE-27 has the disadvantage of being difficult to apply in retrospective studies^14^. For this reason, we could not use the ACE-27 for comparison with the OHNCI in this study.

The magnitude of a comorbid condition is dependent on its prevalence, its mortality, and its influence on treatment selection. The diseases that occur concomitantly with HNSCC vary considerably between different countries. Cerebrovascular disease, chronic pulmonary disease, peptic ulcer disease, and diabetes were the most common comorbidities of patients with HNSCC in a population-based study in Denmark^2^. The most prevalent comorbidity in a cohort of patients with HNSCC from the Netherlands was cardiovascular disease, followed by pulmonary disease^1^. In the cohort used to establish the WUHNCI, pulmonary disease, controlled cancer, diabetes, and myocardial infarction were prevalent comorbidities^3^. In contrast to other regions of the world, synchronous and metachronous SPMs were frequently observed in Japan^4, 15^. Specifically, OC was the most frequent SPM. The high prevalence of OC is relatively unique to Japan. These findings prompted us to develop a new comorbidity index for patients with HNSCC in Japan.

In the present study, we identified SPMs, connective tissue disease, and dementia as independent prognostic factors for patients with HNSCC in Japan. By assigning weighted points to each of these comorbidities, we created the OHNCI. The efficacy of the OHNCI was validated using an independent data set. The OHNCI proved to be more efficient than previously developed comorbidity indices, at least in a Japanese cohort study.

We identified connective tissue disease as an independent prognostic factor and incorporated the disease as an item in the OHNCI, while other comorbidity indices specific to HNSCC (i.e., the HN-CCI and the WUHNCI) do not include connective tissue disease^2, 3^. Several connective tissue diseases, such as rheumatoid arthritis (RA), systemic lupus erythematosus (SLE), Sjögren’s syndrome, dermatomyositis, polymyositis, and scleroderma, are associated with an increased risk of haematological and solid malignancies^16^. RA represents the most common connective tissue disease, and patients with RA are at an increased risk of lymphoma and LC compared to the general population^17^. The sustained stimulation of immune cells and chronic lung inflammation may cause these malignancies. The treatment for RA may also affect the occurrence of malignancies. Treatment with tumour necrosis factor inhibition for patients with RA increases the risk of skin cancer^18^. In contrast, the increased use of non-steroidal anti-inflammatory drugs for RA may reduce the risk of colorectal cancer^17^. SLE is another common connective tissue disease whose association with the incidence of cancer has been extensively investigated. Patients with SLE are at an increased risk of developing haematological and solid malignancies, including HNC, LC, and OC^19, 20^. The aetiology of malignancies arising in patients with SLE has yet to be determined. However, the use of immunosuppressant drugs may at least partially explain the occurrence of malignancies. Furthermore, Asian patients with SLE are at an increased risk of malignancies compared to their European and American counterparts^20^. This difference in ethnicity may have influenced the development of our new comorbidity index.

There are several potential explanations for the poor prognosis of HNSCC patients with connective tissue diseases. First, connective tissue diseases by themselves are sometimes life-threatening conditions. Second, the presence of connective tissue disease often limits the treatment options for HNSCC. The majority of radiation oncologists will not treat patients with connective tissue diseases, owing to severe toxicities, although evidence is lacking to suggest that irradiation is an absolute contraindication for these patients^21^. In addition, impaired organ function in patients with connective tissue diseases may restrict the treatment options for HNSCC. Third, tumour necrosis factor inhibition treatment may increase the recurrence of HNSCC, resulting in a poor prognosis^22^. However, results contradictory to this assumption have been demonstrated in a previous cohort study^23^. Fourth, other malignancies associated with connective tissue diseases may lead to poorer prognoses.

Dementia was a strong prognostic factor for patients with HNSCC in this study. Previous studies revealed that elderly cancer patients with an impaired cognitive status were likely to present with a more advanced stage of cancer at diagnosis^24^. Cancer treatment for patients with dementia is challenging because even minor medical activity requires great effort for both health care providers and patients. Sometimes, families and caregivers will refuse an aggressive cancer treatment. Consequently, those patients are less likely to receive definitive therapy and are at an increased risk of cancer-related mortality^25–27^. Moreover, a previous study has demonstrated that excess mortality of those patients was primarily from non-cancer-related rather than cancer-related causes, partly because they have a greater number of other comorbidities compared to patients without dementia^24^.

SPMs are frequently observed in patients with HNSCC. Their incidence rates and distribution of anatomic sites vary considerably between studies, due to differences in the follow-up duration, the definition of a SPM, screening methods, and the study population^28–30^. In a population-based study in America, the incidence rate of synchronous cancers was 3.0% and the actuarial SPM incidence rate at 25 years from the diagnosis of the index cancer was as high as 61.0%^28^. The most common SPM in this study was LC, followed by HNC. Patients with SPMs in the lungs, oesophagus, and head and neck region were associated with a poorer prognosis compared to those with SPMs in other sites. These findings prompted us to investigate the impact of SPMs according to their anatomic sites, although other comorbidity indices have incorporated SPMs as an item without distinction of their anatomic sites^2, 3, 7, 8, 10, 31^. In our cohort, OC, HNC, and GC represented the most frequently diagnosed SPMs. Consistent with the previous report^28^, SPMs in the oesophagus, lungs, and head and neck region were identified as independent prognostic factors in the present study.

There are several limitations to our study. First, this was a retrospective study. We retrospectively obtained comorbidity data from patients’ medical records. Systematic documentation of a patient’s comorbidities was not enforced. Therefore, the accuracy of the descriptions was dependent on individual physicians. Furthermore, data for drinking and smoking habits were lacking in 5% of cases in the training set and in 30% in the validation set. Therefore, we could not include them in the multivariate model. More importantly, we could not include human papilloma virus (HPV) status in the analyses owing to a lack of data. HPV status is a powerful prognostic factor and is associated with comorbidities^32, 33^. However, incorporation of the HPV status might not change the result, because the proportion of the patients with oropharyngeal cancer and the proportion of HPV positive oropharyngeal cancer are small in Japan. Second, the severity of each of the comorbid conditions was not considered as part of the OHNCI. Incorporation of severity, such as in the ACE-27, may improve the prognostic value of the comorbidity index. However, by excluding severity, the OHNCI can easily be applied to retrospective studies using patients’ medical records and population-based studies using administrative data. Third, we included rare comorbidities (e.g., dementia and connective tissue disease) as items in the OHNCI. The prevalence of dementia in the training set, which was a cohort derived from the teaching hospital, was as low as 0.6% and that of connective tissue disease was as low as 1.1%. Comorbidities with a prevalence of <1.0% were excluded from the development of the HN-CCI and the WUHNCI^2, 3^. However, considering the significant impact of these diseases on the mortality of patients with HNSCC, inclusion of these diseases would appear to be reasonable. Furthermore, the prevalence rates in the validation set, which was a cohort derived from the tertiary care centre, were 3.2% for dementia and 1.3% for connective tissue disease. The prevalence rates of these diseases may be higher in community-based hospitals because patients who are unsuitable for curative treatment would not be referred to tertiary centres. Therefore, these diseases may not be so rare in a real clinical setting. Fourth, only a slight difference in the c-index and AIC were observed between the multivariate model with the OHNCI included and those with other comorbidity indices included. The AIC of the OHNCI model was almost the same as the baseline model. The c-index of the OHNCI model was increased by only 0.006 from the baseline model. As all of these models included age, primary tumour site, Karnofsky PS, and clinical stage as covariates, there was insufficient margin for improvement from the baseline model. Although the OHNCI added only little improvement in accuracy to predict OS, the OHNCI was the only independent prognostic comorbidity index among the indices investigated in this study, and accordingly, the OHNCI is the best index for Japanese patients with HNSCC. Furthermore, considering the sHR of 2.88, the OHNCI would be excellent in the prediction of other cause mortality. We subsequently included only age, sex, PS and OHNCI as covariates into the multivariate model for other cause mortality, because there were 60 patients with other causes of death in the validation set. Further research using a larger data set is required to include other important variables. Lastly, application of the OHNCI is limited. We developed the OHNCI to be best suited for patients with HNSCC in Japan. Therefore, the OHNCI should not be applied to patients with other types of cancer or to patients with HNSCC in countries where the distribution of comorbidities differs from that of Japan. The OHNCI should be used for the evaluation of patients with HNSCC in Japan, and possibly also those in East Asia.

Population aging is a growing concern in many countries. Owing to the increasing elderly population, the comorbidities in cancer patients will become an even greater issue than ever before. Further studies are required to use comorbidity data towards improving the prognosis of patients with HNSCC and the OHNCI represents a useful tool for that purpose.

## Methods

### Ethical statement

The study protocol was approved by the Institutional Review Boards of Osaka University Hospital (Osaka, Japan) and the Osaka General Medical Center (Osaka, Japan). Research was conducted in accordance with the 1964 Declaration of Helsinki and its later amendments. Given the retrospective nature of the study, the need for informed consent was waived by the institutional review boards in accordance with the ethical guidelines for epidemiological research formulated by the Ministry of Health, Labour and Welfare of Japan.

### Patients and data extraction in the training set

The medical records of all previously untreated HNSCC patients who were treated at Osaka University Hospital (Osaka, Japan) between January 2004 and January 2013 were retrospectively reviewed. The inclusion criteria were histologically proven HNSCC and no previous treatment for HNSCC. The exclusion criteria were lost to follow-up within 6 months, and insufficient staging or comorbidity data in the patients’ medical records. The Department’s Cancer Registry system identified 752 consecutive patients with histologically confirmed HNSCC. Nine patients (1.2%) were excluded because of a short follow-up period and 35 patients (4.7%) were excluded because of insufficient clinical data. Finally, 698 patients were included in the training set. The clinicopathological characteristics and comorbidity data of the resultant 698 patients were used to assess the impact of individual comorbidities on HNSCC prognosis. Clinical stage was determined using the seventh edition of the Union for International Cancer Control TNM staging system^34^. Patient PS was determined with Karnofsky PS, and classified into three groups, namely good: 100–80, moderate: 70–50, and poor: 40–0. The initial workup of these patients included contrast-enhanced computed tomography of the neck, 18F-fluorodeoxyglucose positron emission tomography with or without computed tomography, and upper gastrointestinal endoscopy. The index cancer was defined as the cancer having been registered in our Cancer Registry. SPMs were classified as either synchronous or metachronous. All SPMs diagnosed within 6 months of the diagnosis of the index cancer were defined as synchronous SPMs. Among the SPMs diagnosed prior to the 6-month period, SPMs under treatment and SPMs with detectable disease were defined as synchronous SPMs. The remaining SPMs, specifically, SPMs diagnosed prior to the 6-month period, those treated with curative intent, and those without residual disease at the time of diagnosis of the index cancer were defined as metachronous SPMs. Overall survival, index cancer related-, and other cause mortalities were defined as time from the index cancer diagnosis to death of any cause, time from the index cancer diagnosis to death from the index cancer, and time from the index cancer diagnosis to death from causes other than the index cancer, respectively.

Effect of individual comorbidities on overall survival. We investigated the survival rates of patients with or without each comorbid condition in the training set. Survival rates were estimated using the Kaplan-Meier method and compared using the Wilcoxon test.

### Development of the Osaka head and neck comorbidity index

Multivariate analysis was performed using a Cox proportional hazards model. To select predictors for OS, a stepwise forward selection method based on AIC was used. Covariates included in the initial model were age, sex, primary tumour site, clinical stage, PS, and all comorbid conditions listed in Table 2. Comorbidities selected in the final model were considered as independent prognostic factors and were included in the OHNCI. Weightings for the selected comorbidities were determined according to the coefficients of each comorbid condition. The OHNCI score was calculated as the sum of the weightings of each comorbid condition.

### Validation of the Osaka head and neck comorbidity index

We employed an independent data set for validating the OHNCI as a prognostic factor for patients with HNSCC. Specifically, we searched the Cancer Registry at Osaka General Medical Center (Osaka, Japan) to identify patients with HNSCC who were treated between 2006 and 2013. In total, 498 consecutive histologically confirmed HNSCC patients with clinical staging were identified. Of these, 22 patients (4.4%) were excluded because of a short follow-up period of <6 months and 13 patients (2.6%) were excluded because of insufficient clinical data. Finally, 463 patients were selected for analysis.

The data of these 463 patients were used to validate the prognostic capability of the OHNCI. The other comorbidity indices investigated in this study include the CCI^7^, updated CCI^11^, the HN-CCI^2^, and the WUHNCI^3^. Patients in the validation set were divided into three groups for each comorbidity indices according to previous studies^3, 7, 35^. First, OS rates were estimated, according to comorbidity status, using the Kaplan-Meier method and compared using the Wilcoxon test. To investigate the association between the elevated scores of each comorbidity index and the occurrence of death, a Cochran-Armitage test for trends was used. Cumulative incidence rates for index cancer-related mortality and other cause mortality were calculated using non-parametric cumulative incidence functions and compared using the Gray test. Next, Cox proportional hazards models were generated using each comorbidity index as a covariate. Other covariates used included sex, age, primary tumour site, PS, and clinical stage. The HR for mortality, according to comorbidity status, was calculated and the predictive performance of the model was assessed using the c-index and AIC. A Fine and Gray subdistribution hazard model was used to estimate the subdistribution HR for other cause mortality.

### Statistical analyses

Sample size was not determined statistically. All available data with adequate follow-up in the registries were used to maximise the generalisability of the results. Patients with missing data were omitted from this study, so that no imputation method was used. The chi-square test was used to assess the associations between categorical variables and the Mann-Whitney U test was used to assess the associations between categorical and continuous variables. Survival rates were estimated using the Kaplan-Meier method and compared using the Wilcoxon test. Cumulative incidence rates for cancer-related mortality and non-cancer-related mortality were calculated using non-parametric cumulative incidence functions and compared using the Gray test. Multivariate analyses were performed using a Cox proportional hazards model and a Fine and Gray subdistribution hazard model. P < 0.05 was considered statistically significant. Statistical analyses were conducted using R (The R Foundation for Statistical Computing, Vienna, Austria) and JMP Pro software version 12 (SAS Institute Japan, Tokyo, Japan).

### Data availability statement

The datasets generated during and/or analysed during the current study are available from the corresponding author on reasonable request.
